# 

**Published:** 1899-05-27

**Authors:** 


**Notices of Births, Deaths, and Marriages** are inserted in this column at a charge of 2s. 6d. for an announcement not exceeding four lines.

**Notice to Advertisers and Subscribers.**

All Advertisements, Orders for Copies of The Hospital, and Business Communications should be addressed to **THE MANAGER (Not to the Editor)**, The Hospital, The Hospital Building, 28 & 29, Southampton Street, Strand, London, W.C.

The Cost of Subscription, post free, is as follows:—Per week, 2½d.; per quarter, 2s. 8d.; per half-year, 5s. 3d.; per annum, 10s. 6d. Foreign:—Per annum, 15s. 2d., payable, in all cases, in advance.

**NOTICE.—Vol. XXV. of "The Hospital" (October, 1898, to March, 1899), in handsome cloth binding, is now on sale at the Office of this Journal, price 7s. 6d. Cases for binding the Numbers contained in this Volume 1s. 6d. Index to Vol. XXV., 2½d., post free.**

**The Monthly Part for June will be ready on June 1st. Price 6d., by post 8d.**

### BOOKS RECEIVED.

CASSELL AND CO.

"Hygiene and Public Health." By B. Arthur Whitelegge, M.D., B.Sc., F.R.C.P., D.P.H.

"Materia Medica and Therapeutics." By J. Mitchell Bruce, M.D., F.R.C.P.

ADAM AND CHARLES BLACK.

"Milk: Its Nature and Composition." By C. M. Aikman, M.A., D.Sc.

J. R. WILLIAMS AND CO., LIVERPOOL.

"Report on the Health of the City of Liverpool During 1898." By E. W. Hope, M.D., D.Sc., Medical Officer of Health.

J. AND A. CHURCHILL.

"A Short Practice of Midwifery, Embodying the Treatment Adopted in the Rotunda Hospital, Liverpool." By Henry Jellett, M.D., F.R.C.P.I. With a preface by W. J. Smyly. M.D., F.R.C.P.I.

JOHN BALE, SONS, AND DANIELSSON.

"The Modern Doctrine of Bacteriology, with Special Reference to Gynæcology." By George Granville Bantock, M.D., F.R.C.S.E.

"The Nurses' Clinical Case Book."

ROBERTSON AND GRAY, COVENTRY.

"Annual Report on the Health of the City of Coventry." By E. Hugh Snell, M.D., B.Sc., Medical Officer of Health.

S. HARVEY, EARLSTOWN.

"Annual Report to the Haydock Urban District Council." By T. E. Hayward, M.B., Medical Officer of Health.

JOHN WRIGHT AND CO.

"Golden Rules of Medical Practice." By Arthur Henry Evans, M.D., B.Sc., F.R.C.S.

HENRY J. GLAISHER.

"Clinical Lectures on Neurasthenia." By Thomas D. Savill, M.D.

A. AND C. BLACK.

"Milk: Its Nature and Composition." By C. M. Aikman.

### Scraps and Gleanings.

Lord Wantage, the president of the Royal Berkshire Hospital, has given a sum of £1,000 to the hospital.

The executors of the late Mr. Mainwaring have given to the Liverpool Hospital for Consumption a sum of £250.

The trustees of Smith's Charity have sent a donation of £100 to the Royal Hospital for Diseases of the Chest, City Road.

The amount of the tender that has been accepted for the Jubilee wing extension of the Nottingham General Hospital is £23,786.

During 1898 1,034 patients were admitted to the Convalescent Home at Linden, Blackrock, Dublin. The year's expenditure was £2,750, leaving a deficit of income of £316.

A small committee has been appointed to consider the advisability of the City Hospital Committee of Aberdeen Town Council purchasing the Aberdeen public baths, it being thought that the building might be required in connection with the hospital.

The first annual dinner of the North-Eastern Hospital Children's Aid Society proved a great success, realising no less a sum than £286 odd. This society appears to be doing excellent work, and the result of the year's working was that a sum of £350 was handed to the hospital; the expenditure for the year being only £3 5s. 9d.

It has been decided, in consequence of the non-success from a financial point of view of last year's effort, that no fête shall be held this year by the Crediton Friendly Societies in aid of the Hospital Saturday Fund, and to limit the effort of the Friendly Societies to a church parade only, which is to be held on the Sunday preceding the August Bank Holiday.

Although considerable retrenchment has been effected through closing a number of beds in the Royal Berkshire Hospital, the committee are still in urgent need of funds. It is estimated that the amount which is needed to carry on the work of the hospital efficiently is £40,000 by donations to form a capital fund, and an increase in the subscriptions of not less than £600.

The funds at the command of the Sister Dora Hospital at Milfor are only sufficient for the next eight months.

A native of Chorley has expressed a desire to expend about £3,000 in the erection of a new wing to the Chorley Hospital, which was first opened in 1893.

The new nurses' dormitories which have just been completed at Sussex County Hospital provide accommodation for 50 nurses, each having a separate bed-room.

The commemoration stone of the Queen's Jubilee Cottage Hospital at Hanwell is to be laid by the Countess of Jersey in June. The sum of about £425 is still required to complete and furnish the hospital.

Lord George Hamilton is appealing for funds to cover the expenses of the transference of the Ealing Provident Dispensary to a new and suitable house on Ealing Green. A sum of £1,200 will be required for this purpose.

An anonymous donor, a lady, has offered a donation of £1,000 to endow a bed in the Glasgow Samaritan Hospital for Women, provided two other donors come forward with a similar donation before the beginning of next year.

At the closing of the Trades and Industrial Exhibition which has been held for two months at Bingley Hall, Birmingham, a sum of £210 was handed over to the Queen's Hospital, this amount being 5 per cent. of the gross receipts for admission to the exhibition. The promoters of the exhibition have for some years made it a rule to give this percentage to one of the local charities.

In the wreck of the "Mohegan" off the Manacles last autumn a number of survivors were temporarily housed in the Royal Sailors' Home at Falmouth, which has again rendered good service in this direction in the wreck of the "Paris." The committee should, therefore, find a ready response to the appeal they issue for further funds to enable the requisite additions and improvements to be made.

